# Multiple synchronous gliomas of distinctly different grades

**DOI:** 10.4103/2152-7806.69376

**Published:** 2010-09-16

**Authors:** Patrick J. Kelly

**Affiliations:** Department of Neurological Surgery, NYU School of Medicine, 530 First Ave, Suite 8R, New York, NY - 100 16, USA

It is not surprising that a multicentric glioma would exhibit two different cellular phenotypes. In contrast to what most of us believed about the oncogenesis of gliomas – that gliomas resulted from the dedifferentiation of mature cells – it is now becoming increasingly clear that gliomas evolve from neural stems cells and/or the progenitors that arise from these stem cells.[3] Neural stem cells can become neurons, astrocytes or oligodendrocytes and/or progenitors such as the O2A progenitor that become either type II astrocytes or oligodedrocytes. It is also clear that following embryogenesis, viable multipotential stem cells persist in stem cell clusters even in the adult mammalian brain. In a conducive *in-vitro* or *in-vivo* microenvironment, cells in these stem cell clusters can evolve into gliomas of mixed cell types that contain primitive astrocytes, oligodendroglial cells and neurons.

What is surprising is that cases, such as the one reported in this article, seem to be relatively rare. This may be because most of these multicentric gliomas remain subclinical, microscopic and limited to small populations of several hundred cells that remain in a steady-state balance of mitosis and apoptosis. Occasionally, the balance of apoptosis/mitosis is upset in one of these colonies and one of these lesions then progresses to a clinically significant glioma.

Low grade gliomas become high grade gliomas whose clinical phenotype is at the mercy of the subpopulation with the highest mitotic rate. Cellular phenotypic clones with the highest mitotic rate will eventually overwhelm the tumor that is then classified as that phenotype (e.g. astrocytoma) at diagnosis and evolve into the malignant tumor that kills the patient.

Perhaps the apparent “rarity” of distinct multicentric gliomas is due to the uncommon simultaneity of the transformation of two tumor stem cell clusters from “steady state” indolent, clinically silent lesions into clinically significant tumors within a finite epoch. During this time, one of the lesions destroys enough brain tissue or results in a significant enough mass to cause the symptoms that lead to the clinical detection and eventual diagnosis. Sadly, when most gliomas are diagnosed, they are too advanced to be curable by current therapies.

In fact, clinically silent gliomas are much more common than the US incidence rate of about 20,000 cases per year would suggest. Imaging based screening studies done on large populations have indicated a prevalence of about 3 cases per 1000 population.[1,2,4] This is not a huge number, of course. Nonetheless, a US population of 310,000,000 people contains over 900,000 individuals harboring asymptomatic gliomas that are large enough to be detected by imaging methods. We know how many of these become symptomatic each year. We do not know how many will become symptomatic in a lifetime.
